# Relative effects of LDL-C on ischemic stroke and coronary disease: A Mendelian randomization study

**DOI:** 10.1212/WNL.0000000000007091

**Published:** 2019-03-12

**Authors:** Elsa Valdes-Marquez, Sarah Parish, Robert Clarke, Traiani Stari, Bradford B. Worrall, METASTROKE Consortium of the ISGC,, Jemma C. Hopewell

**Affiliations:** From the Clinical Trial Service Unit and Epidemiological Studies Unit (E.V.-M., S.P., R.C., T.S., J.C.H.) and MRC Population Health Research Unit (S.P.), Nuffield Department of Population Health, University of Oxford, UK; and Departments of Neurology and Public Health Sciences (B.B.W.), University of Virginia School of Medicine, Charlottesville, VA.

## Abstract

**Objective:**

To examine the causal relevance of lifelong differences in low-density lipoprotein cholesterol (LDL-C) for ischemic stroke (IS) relative to that for coronary heart disease (CHD) using a Mendelian randomization approach.

**Methods:**

We undertook a 2-sample Mendelian randomization, based on summary data, to estimate the causal relevance of LDL-C for risk of IS and CHD. Information from 62 independent genetic variants with genome-wide significant effects on LDL-C levels was used to estimate the causal effects of LDL-C for IS and IS subtypes (based on 12,389 IS cases from METASTROKE) and for CHD (based on 60,801 cases from CARDIoGRAMplusC4D). We then assessed the effects of LDL-C on IS and CHD for heterogeneity.

**Results:**

A 1 mmol/L higher genetically determined LDL-C was associated with a 50% higher risk of CHD (odds ratio [OR] 1.49, 95% confidence interval [CI] 1.32−1.68, *p* = 1.1 × 10^−8^). By contrast, the causal effect of LDL-C was much weaker for IS (OR 1.12, 95% CI 0.96−1.30, *p* = 0.14; *p* for heterogeneity = 2.6 × 10^−3^) and, in particular, for cardioembolic stroke (OR 1.06, 95% CI 0.84−1.33, *p* = 0.64; *p* for heterogeneity = 8.6 × 10^−3^) when compared with that for CHD.

**Conclusions:**

In contrast with the consistent effects of LDL-C-lowering therapies on IS and CHD, genetic variants that confer lifelong LDL-C differences show a weaker effect on IS than on CHD. The relevance of etiologically distinct IS subtypes may contribute to the differences observed.

Stroke is a heterogeneous collection of clinically related but distinct disorders, with ischemic stroke (IS) representing 70%–90% of all strokes.^1,2^ Different IS subtypes have distinct underlying pathologies that likely reflect differences in the importance of underlying risk factors, such as hypertension and dyslipidemia, as well as in genetic determinants.^3–6^

Randomized trials of statin therapy have demonstrated that lowering low-density lipoprotein cholesterol (LDL-C) by 1 mmol/L reduces the risk of both IS and coronary heart disease (CHD) by about 20%.^7^ Other LDL-C-lowering therapies, such as ezetimibe and PCSK9 inhibitors, also yield comparable reductions in IS and CHD risk.^8,9^ In contrast, observational studies have found stronger effects of LDL-C on CHD than on IS,^10^ and potential heterogeneity in the effects of cholesterol on different IS subtypes.^6^ Therefore, further evidence is needed to determine whether LDL-C has comparable causal consequences for IS and CHD.

Mendelian randomization avoids many of the potential biases of observational studies, such as reverse causation and confounding. Mendelian randomization studies use genetic variants as instrumental variables that reflect lifelong differences in exposure to a risk factor, in order to examine its causal relevance for an outcome of interest. However, Mendelian randomization can be sensitive to pleiotropy, in which genetic variants are associated with multiple risk factors on different biological pathways. Mendelian randomization studies have been widely used to examine risk factors for CHD,^11–14^ but studies of IS have been limited.^15–17^

The present Mendelian randomization study examines the causal relevance of LDL-C for IS and compares it with that for CHD.

## Methods

### Study populations

We obtained genome-wide association estimates for LDL-C, high-density lipoprotein cholesterol (HDL-C), and triglycerides from the Global Lipids Genetics Consortium (GLGC), based on up to 188,577 participants of European ancestry.^18^ The effects of these genetic variants on CHD were examined in the CARDIoGRAMPlusC4D Consortium including up to 60,801 CHD cases and 123,504 controls from 48 studies of predominantly European ancestry.^19^ Similarly, the effects on IS and IS subtypes were examined in METASTROKE, a collaboration of the International Stroke Genetics Consortium, which brings together genome-wide data on a total of 12,389 IS cases and 62,004 controls of European ancestry from across 15 studies.^20^ The majority of IS cases had brain imaging confirmation. Approximately 50% of cases had IS subtype information (2,365 cardioembolic, 2,167 large artery, and 1,894 small vessel stroke cases) based on Trial of Org 10172 in Acute Stroke Treatment classifications.^21^ Additional phenotype descriptions and details of individual studies, including data collection and genetic data quality control procedures, are reported elsewhere.^20^

### Standard protocol approvals, registrations, and patient consents

Each study included in the consortia was approved by an institutional review board, and all patients provided informed consent.

### Selection of LDL-C associated genetic variants

We selected genetic variants with genome-wide significant (*p* < 5 × 10^−8^) associations with LDL-C in the GLGC meta-analysis and that were available in both the CARDIoGRAMplusC4D and METASTROKE datasets. Of these 2,243 genetic variants, we identified 99 independent variants (*r*^2^ < 0.01 within ± 1,000 kb) using the clumping method implemented in PLINK1.9 and 1,000 Genomes Project Phase 3 (EUR) reference population.^22,23^ Finally, to identify variants with LDL-C-specific lipid effects (and avoid pleiotropy through effects on other lipid pathways), we excluded the 37 variants with significant effects on HDL-C or triglycerides (*p* < 0.0005 based on Bonferroni correction for 99 variants). Hence, the primary analyses were restricted to the 62 variants with LDL-specific effects, with sensitivity analyses performed using all 99 variants that were independently associated with LDL-C (table e-1; doi.org/10.5061/dryad.8076h3r).^18^

### Statistical analysis

Per-allele effects for LDL-C were extracted from GLGC and converted from the published SD units to mmol/L (1 SD unit equating to ∼1 mmol/L). Per-allele effects of the variants on CHD were taken from CARDIoGRAMplusC4D^19^ and on IS (and IS subtypes) from METASTROKE.^20^ To account for multiple testing, we used a predefined *p* value threshold of *p* < 0.0005 to indicate statistically significant associations of individual variants with risk of disease, and report all effects with respect to the LDL-C increasing allele unless otherwise stated. The percentage of variance explained in LDL-C was estimated by 2 × (effect on LDL-C in SD units)^2^ × minor allele frequency × (1 − minor allele frequency),^24^ and power calculations for *p* < 0.01 were estimated from the variance explained and sample size.^25^

Causal effects on disease outcomes per 1 mmol/L genetically higher LDL-C were estimated using the random-effects inverse-variance weighted method for summarized data (in which all genetic variants included are assumed to be valid instrumental variables).^26^ To account for the multiple outcomes tested, a predefined *p* value threshold of *p* < 0.01 was used to indicate statistically significant causal associations. We conducted methodologic sensitivity analyses^27,28^ using the Mendelian randomization–Egger (MR-Egger) method (in which all genetic variants are permitted to be invalid instrumental variables, provided that the pleiotropic and risk factor effects of the variants are independently distributed—known as the instrument strength independent of direct effect assumption—and allows assessment of directional pleiotropic bias)^29,30^; the weighted median method (in which 50% of the genetic variants are permitted to be invalid instrumental variables)^31^ and the multivariate method (in which potentially pleiotropic effects on HDL-C and triglycerides are allowed for by including terms for each lipid (table e-1; doi.org/10.5061/dryad.8076h3r) in the estimation of the causal effects, while fixing the intercept term as zero).^32^ The Mendelian randomization–Pleiotropy Residual Sum and Outlier (MR-PRESSO) method (which performs a pleiotropy residual sum and outlier test and allows detection and correction of pleiotropy by outlier removal) was also used to evaluate potential pleiotropy and identify outlying variants that were then excluded from the analyses.^33^ Heterogeneity between the causal effects of individual variants, as well as comparisons between the causal effects of LDL-C on CHD vs IS (and IS subtypes), were tested using the Cochran Q statistic.^27^ All statistical analyses were performed in SAS v9.3 or R v3.4.3.

### Data availability

The data included in the reported analyses have been made publicly available (also see Acknowledgement for additional details on data access).

## Results

### Effects of LDL-C genetic variants on CHD, IS, and IS subtypes

**Figure 1:**
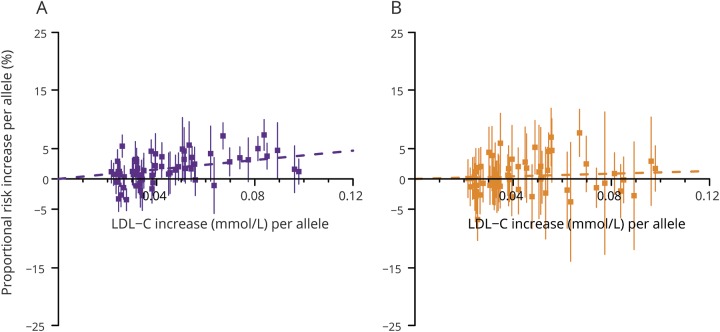
Effects of genetic variants on coronary heart disease and ischemic stroke risk vs low-density lipoprotein cholesterol (LDL-C) levels Figures are shown separately for (A) coronary heart disease and (B) ischemic stroke. Effects of the 62 individual genetic variants in the primary analysis are shown per LDL-C increasing allele.

The effects of the 62 individual genetic variants on LDL-C levels varied by 5-fold, ranging from 0.02 mmol/L to 0.10 mmol/L per allele (table e-1; doi.org/10.5061/dryad.8076h3r), and in combination explained about 4% of the variance in LDL-C. Despite limited power to detect risk associations with individual variants, 8 variants were associated with CHD and 2 with IS (*p* < 0.0005; table e-2; doi.org/10.5061/dryad.8076h3r). The effects of the 62 variants on IS and IS subtypes were consistently weaker than their effects on CHD (figure 1 and table e-3 and figures e-1 and e-2; doi.org/10.5061/dryad.8076h3r).

### Causal effects of LDL-C on CHD, IS, and IS subtypes

**Figure 2:**
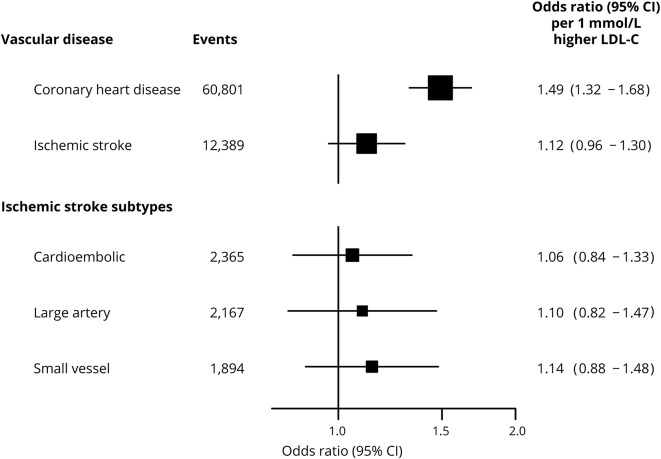
Effects of genetically determined low-density lipoprotein cholesterol (LDL-C) on vascular disease and ischemic stroke subtypes Causal estimates are based on 62 variants associated with LDL-C in the primary analysis. Odds ratio and 95% confidence intervals (95% CIs) are provided for vascular disease (coronary heart disease and ischemic stroke) and ischemic stroke subtypes per 1 mmol/L higher genetically determined LDL-C.

Genetically determined LDL-C was associated with about a 50% higher risk of CHD per 1 mmol/L (odds ratio [OR] 1.49, 95% confidence interval [CI] 1.32 to 1.68; *p* = 1.1 × 10^−8^) but, by contrast, had no effect on IS (OR 1.12, 95% CI 0.96 to 1.30; *p* = 0.14). There were also no effects of genetically determined LDL-C on any of the individual subtypes of IS (figure 2).

The effect of LDL-C on IS was weaker than that on CHD (*p* for heterogeneity = 2.6 × 10^−3^), and in particular on cardioembolic stroke (*p* for heterogeneity = 8.6 × 10^−3^), whereas the effects of LDL-C on large artery stroke and small vessel stroke were compatible with the magnitude of the effect observed for CHD (*p* for heterogeneity = 0.05 and 0.06, respectively; figure 2). Furthermore, given >99% power to detect a 30% increase in risk of IS at *p* < 0.01 (equivalent to the lower limit of the CI for CHD), these analyses can exclude a causal effect of LDL-C on total IS of the same magnitude as on CHD. However, given comparatively little power (<50%) to detect 30% causal effects for separate IS subtypes, comparable effects of LDL-C on CHD and particular IS subtypes cannot be excluded.

### Sensitivity analyses

Sensitivity analyses were undertaken based on an instrument including 99 LDL-C-associated variants (of which 37 were also associated with HDL-C or triglycerides). This genetic instrument explained 11% of the variance in LDL-C, and was strongly influenced by the *TOMM40/APOE* locus, which represented ∼2% of the variance in LDL-C. The estimates of the LDL-C causal effects on disease outcomes did not differ meaningfully from the primary analysis involving 62 variants with LDL-C-specific effects (figure e-3; doi.org/10.5061/dryad.8076h3r). However, they were slightly weaker, 1.05 (95% CI 0.96 to 1.15) vs 1.12 (95% CI 0.96 to 1.30) for IS per 1 mmol/L higher LDL-C, and showed greater heterogeneity between individual variant causal effects than the primary analysis instrument (*p* = 1.0 × 10^−5^ vs *p* = 2.5 × 10^−3^). A similar pattern was also observed when comparing the causal effects of the different genetic instruments for CHD.

In the primary analyses, the LDL-C causal effect estimates for CHD and IS across genetic variants obtained by the inverse-variance weighted approach were consistent with those obtained by the weighted median and multivariate Mendelian randomization methods (table 1). There was no evidence of directional pleiotropy for either CHD (bias = -0.012, p = 0.07) or IS (bias = -0.014, *p* = 0.08). The causal estimates from the MR-Egger analysis were greater than those obtained by other methods. However, MR-Egger results should be interpreted with caution due to potential bias from outlying variants. The exclusion of outlying variants identified by MR-PRESSO reduced the causal estimates from MR-Egger, as well as the estimates of pleiotropic bias (bias = -0.006, *p* = 0.23 for CHD and bias = -0.008, *p* = 0.26 for IS). The heterogeneity between variants was also attenuated after making these exclusions (*p* = 1.7 × 10^−9^ vs 1.2 × 10^−5^ for CHD and *p* = 2.5 × 10^−3^ vs 0.18 for IS). Based on the 99-variant instrument, estimates were consistent across all the methods explored and there was no evidence of directional pleiotropy.

**Table 1:**
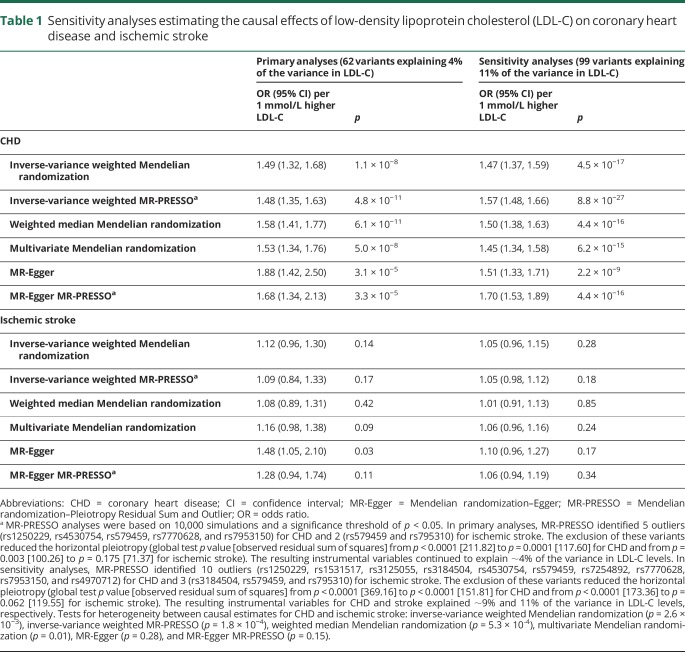
Sensitivity analyses estimating the causal effects of low-density lipoprotein cholesterol (LDL-C) on coronary heart disease and ischemic stroke

Evidence of heterogeneity between the causal effects of LDL-C on CHD vs IS was consistent for all analysis approaches, with the exception of MR-Egger in the primary analyses and without exception for the 99-variant sensitivity analysis demonstrating weaker effects of genetically determined LDL-C on IS than on CHD (table 1).

### Comparing observational, randomized, and genetic evidence

**Figure 3:**
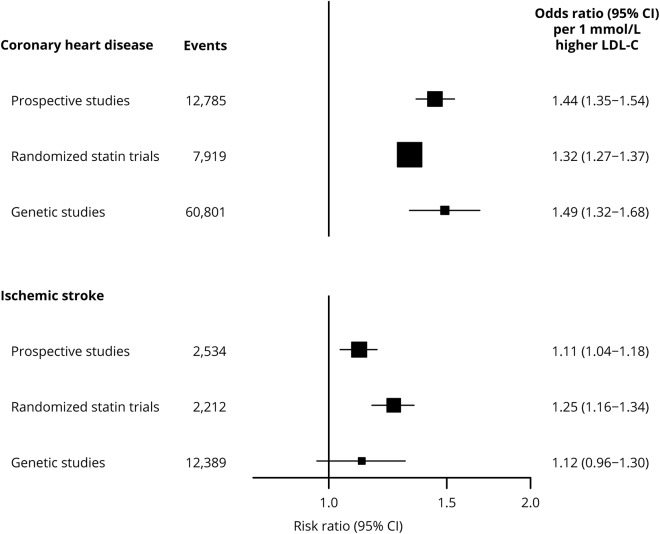
Effects of low-density lipoprotein cholesterol (LDL-C) on vascular disease in prospective studies, randomized statin trials, and genetic studies Genetic effect of LDL-C on disease was estimated based on 62 variants associated with LDL-C (see primary analysis methods). Estimates from prospective studies are shown for usual levels of non-high-density lipoprotein cholesterol.^10^ Estimates from randomized statin trial for coronary heart disease are based on major coronary events (coronary death or nonfatal myocardial infarction).^7^ Estimates from genetic studies are taken from figure 2. CI = confidence interval.

The effects of genetically determined LDL-C (per 1 mmol/L higher) on CHD and IS in the present study were similar to the corresponding effects reported for equivalent LDL-C changes in observational studies (figure 3).^7,10^ As observed in the genetic data, the observational associations of LDL-C with stroke were weaker than those with CHD (*p* = 3.2 × 10^−8^). In contrast, there was no such heterogeneity between the effects observed in the statin trials (*p* = 0.20).

## Discussion

This Mendelian randomization study provides a large-scale comparison of the lifelong effects of LDL-C on risk of vascular disease, and demonstrates that genetically determined LDL-C has a weaker effect on IS than on CHD. Furthermore, these results were robust to the selection of LDL-C genetic variants used to estimate the causal effect as well as to different statistical approaches to Mendelian randomization analyses.

Observational evidence suggests that in addition to a differential effect of cholesterol on IS and hemorrhagic stroke, the effect of cholesterol on IS varies by subtype.^6,34^ In contrast, the Stroke Prevention by Aggressive Reduction in Cholesterol Levels (SPARCL)^35^ trial reported that atorvastatin effectively prevented recurrent stroke (independently of the subtype of the previous stroke), but did not indicate that statins had differential effects on specific IS subtypes. However, genetic data from the SiGN study suggested a somewhat stronger effect of LDL-C on large artery stroke than on other IS subtypes.^15^ The present genetic study, which includes ∼7,000 independent IS cases not previously reported in the SiGN study, showed a nonsignificant 12% higher risk on IS per 1 mmol/L genetically determined LDL-C, and relatively consistent effects of LDL-C across IS subtypes. However, this analysis had limited power to assess the causal effects of LDL-C on specific IS subtypes and on the compatibility with the effect on CHD. Furthermore, differences in the ethnicity of participants (SiGN included some non-European participants), in the instrumental variables used and clumping criteria (in which the present study was more stringent to avoid overweighting), as well as unknown differences in vascular risk factor distributions may contribute to discrepancies between the studies. Thus, given the biological plausibility of differential effects of LDL-C on different IS subtypes (and previous evidence that genetic determinants of stroke are commonly subtype-specific^20^), larger scale Mendelian randomization studies are still needed to clarify the lifelong effects of LDL-C on etiologically distinct IS subtypes. In addition, IS subtype information is needed in large-scale randomized trials of LDL-modifying therapies to directly assess their effects on different subtypes of IS.

The analogy between Mendelian randomization and randomized clinical trials is commonly used. However, Mendelian randomization studies examine the lifelong cumulative effects of a risk factor, while clinical trials examine the short-term effect of a therapy. Consequently, the effect estimates from Mendelian randomization studies and randomized trials are not expected to be directly comparable. Mendelian randomization can assess the causal relevance of risk factors and help to anticipate relative effects of therapies on different disease outcomes, by studying genetic variants that have direct effects on a risk factor or that mimic therapeutic interventions, and by exploring the effects for one outcome relative to another, as in the present study.^36^

Genetic variants that affect LDL-C levels via various biological pathways were combined in the analyses described to provide a strong instrument for LDL-C, under the assumption that LDL-C has consistent effects across all these mechanisms. However, genetic studies examining the effects of specific therapeutic targets that affect LDL-C and other biomarkers are also important for drug target evaluation. Recent studies examining instruments based on specific genes that mimic the effects of lipid-modifying therapies, such as *PCSK9*, *HMGCR*, and *NPC1L1*, have shown weaker effects on IS than on CHD, but also suggest that the different pathways involved may affect stroke subtypes differentially.^15,37,38^ A study of the combined effects of *CETP* and *HMGCR* has also suggested that the benefits of lowering LDL-C may depend on the reduction in apoB-containing lipoprotein particles.^39^

The effects of LDL-C on IS were comparable to those on CHD in randomized trials of statin therapy, but were smaller for IS than for CHD in this genetic study (figure 3). Clinical trials of lipid-modifying therapies have typically recruited a high proportion of participants with, or at high risk of, coronary heart disease, and hence such patients are likely to have high levels of atherosclerosis. In the Cholesterol Treatment Trialists' meta-analysis of randomized statin trials, over 50% of participants had established CHD, and 70% had ≥10% 5-year risk of a major vascular event.^40^ By contrast, the majority of METASTROKE IS cases were recruited through acute stroke services or population studies and individuals thus are less likely to have comparable levels of atherosclerotic disease and risk. For example, in a hospital-based cohort of 4,033 stroke patients, only 10% had a history of myocardial infarction.^4^ Consequently, the relative contribution of different risk factors and the resulting distribution of IS subtypes may differ in the METASTROKE and randomized trial participants. A higher proportion of stroke cases in the METASTROKE meta-analysis may be due to non-atherosclerotic risk factors, such as atrial fibrillation, resulting in more cardioembolic strokes. By contrast, IS events in trials are more likely to be due to atherosclerosis resulting in a higher proportion of large artery strokes, for which therapeutic LDL-C lowering effects may have greater relevance. Such factors may also explain the stronger effects of LDL-C in randomized trials than in observational studies.

Etiologic differences in stroke may mean that even modest misclassification of IS could attenuate results, particularly given previous evidence indicating that lower LDL-C levels are associated with higher risks of hemorrhagic stroke.^7^ However, differential relevance of risk factors and pathways for CHD and IS as well as differences in patient characteristics between cohorts may explain some of the differences between IS and CHD observed in the present study.

Mendelian randomization analyses avoid many of the biases inherent in observational studies (e.g., confounding and reverse causation). However, such analyses rely on underlying assumptions, for example the validity of the instrument and the untestable MR-Egger INSIDE assumption, and can also suffer from weak instrument bias. To explore the robustness of the analyses, the causal effect of LDL-C on disease outcomes was estimated by various Mendelian randomization methods that relax the instrumental variable validity assumption as well as after removal of outlying variants. The analyses conducted showed no meaningful differences. Furthermore, the estimates from this Mendelian randomization study were consistent with recent reports examining the individual causal effects of LDL-C on IS and on CHD.^13,15,37,41–43^

This study suggests that LDL-C has a substantially weaker causal effect on IS than for CHD, a result that has potential implications for evaluation and development of therapeutic approaches. Additional large-scale genetic studies of IS, particularly with regard to specific IS subtypes and diverse ethnic populations, are needed to further elucidate these relationships. In addition, metabolomic studies may offer additional insights given that different LDL-C subparticles and their comparative pathogenicity for IS and different IS subtypes may be important given previous evidence of differences in the genetic determinants of the different particle sizes.^44^

## Glossary

CHD: coronary heart disease
CI: confidence interval
HDL-C: high-density lipoprotein cholesterol
GLGC: Global Lipids Genetics Consortium
IS: ischemic stroke
LDL-C: low-density lipoprotein cholesterol
MR-Egger: Mendelian randomization–Egger
MR-PRESSO: Mendelian randomization–Pleiotropy Residual Sum and Outlier
OR: odds ratio
